# Pre-fatigue Direct 1RM Testing vs. Post-fatigue Load–Velocity Profiling: Which Better Predicts Maximal Strength in the Deadlift After Resistance Training?

**DOI:** 10.1186/s40798-026-01097-8

**Published:** 2026-08-27

**Authors:** Danica Janicijevic, Deniz Şentürk, Yaodong Gu, Amador García-Ramos

**Affiliations:** 1 Faculty of Sports Science, Ningbo University, Ningbo, China; 2 Research Academy of Human Biomechanics, The Affiliated Hospital of Medical School of Ningbo University, Ningbo University, Ningbo, China; 3 Department of Health Research, Icen Cognis, Carretera General, 32, 38370 La Matanza de Acentejo, Tenerife, Spain; 4 Faculty of Health Sciences, Universidad Tecnológica Atlántico Mediterráneo - UTAMED, Málaga, Spain; 5 Department of Coaching Education, Faculty of Sports Science, Istanbul Gelişim University, 34000 Istanbul, Turkey; 6 Department of Physical Education and Sport, Faculty of Sport Sciences, University of Granada, Granada, Spain; 7 Department of Sports Sciences and Physical Conditioning, Faculty of Education, Universidad Catolica de la Santísima Concepción, Concepción, Chile

**Keywords:** Linear position transducer, One-repetition maximum, Strength training, Testing, Velocity-based training

## Abstract

**Background:**

Resistance training loads are commonly prescribed based on a previously determined 1-repetition maximum (1RM). Whether velocity-based methods better reflect the actual 1RM as fatigue accumulates during the training session than relying on a pre-training 1RM remains unknown.

**Objectives:**

To determine whether pre-training direct 1RM testing or post-training load–velocity (L–v) profiling more accurately estimates hexagonal barbell deadlift (HBD) 1RM following resistance training-induced fatigue.

**Methods:**

Twenty-seven resistance-trained males (HBD 1RM = 176.8 ± 20.9 kg) randomly completed three experimental sessions, each involving full incremental loading tests before and after a 30-min intervention: (i) control (rest), (ii) moderate fatigue (5 sets at 70% 1RM performing half the maximal possible repetitions), or (iii) high fatigue (5 sets at 70% 1RM to failure). Post-session 1RM was estimated using six methods: pre-session 1RM, post-session L–v profile, and four velocity-based methods that used the post-session mean velocity (MV) recorded at 50%, 70%, 80%, and 90% 1RM applied to the pre-session %1RM–MV relationship. Each estimate was then compared with the directly measured post-session 1RM. The study was conducted at Istanbul Gelişim University (Istanbul, Türkiye) between 29 March and 26 April 2024.

**Results:**

The pre-session 1RM (9.5 ± 9.4 kg) and MV50% (− 13.4 ± 13.0 kg) substantially over- and underestimated the actual 1RM, respectively, while smaller errors were observed for the post-session L-v profile (1.9 ± 7.7 kg), MV70% (− 2.8 ± 8.6 kg), MV80% (− 2.5 ± 7.8 kg), and MV90% (− 0.9 ± 5.9 kg). Based on absolute errors, methods were ranked from least to most accurate as: MV50% (15.4 ± 10.5 kg) < pre-session 1RM (9.5 ± 9.4 kg) < MV70% (7.1 ± 5.6 kg) = MV80% (6.5 ± 4.9 kg) = post-session L–v profile (5.9 ± 5.2 kg) < MV90% (4.7 ± 3.7 kg). Notably, pre-session 1RM errors increased with greater fatigue, while those from velocity-based methods remained more stable.

**Conclusions:**

Reliance on a pre-session 1RM may result in systematic overestimation of relative training loads under resistance training-induced fatigue, whereas velocity-based methods appear to provide more accurate prescriptions of training intensity (%1RM).

## Introduction

The 1-repetition maximum (1RM) is the primary reference for individualizing loads in resistance training (RT) programs [1–3]. Its direct assessment is considered the gold standard [4]; however, because this procedure is both time- and effort-demanding, several alternatives have been proposed to estimate it [5–7]. Among these, the most widely adopted approach in recent years relies on measuring lifting velocity against submaximal loads [5], which requires less time and imposes lower physiological stress. It is well-established that systematic strength training elicits progressive increases in 1RM, whereas periods of detraining result in its decline [8–10]. Accordingly, long-term interventions consistently incorporate regular 1RM reassessments—typically every 3–5 weeks—reflecting broad agreement on the necessity of periodic evaluations of maximal dynamic strength to ensure precise load prescription [11, 12]. However, the necessity of short-term evaluations of maximal dynamic strength—whether through direct testing or indirect estimations—to account for daily readiness or acute fatigue remains a matter of debate.

Some coaches and researchers argue that 1RM remains highly stable over short time frames, thereby questioning the practical relevance of frequent reassessments or the use of velocity-based estimation methods. For example, Zourdos et al. [13] reported that the back squat 1RM of three competitive lifters tested across 36 consecutive days exhibited remarkable day-to-day stability (absolute differences: 2.4 ± 1.7%). Similarly, Blumert et al. [14] observed that 24 h of sleep deprivation did not impair 1RM strength despite a marked deterioration in mood state. It should also be noted that the frequently cited notion of high between-day variability in 1RM does not originate from studies directly assessing daily fluctuations in actual 1RM, but rather is a colloquial perception among practitioners inferred from velocity recordings. This distinction is important, as lifting velocity typically shows greater variability than traditional strength measures [15, 16], and because changes in 1RM under stable conditions are generally attributable to the small measurement variability of the test, whereas fatigue-induced reductions reflect true biological changes in maximal strength rather than measurement error. Taken together, these findings suggest that direct 1RM testing may be robust under stable conditions; however, relying on a pre-fatigue 1RM may not accurately reflect maximal strength once fatigue accumulates during RT, potentially leading to inaccurate relative load prescriptions.

In practice, prescribing RT loads based on a previously determined 1RM implicitly assumes that maximal strength remains stable throughout the training session. However, this assumption may not hold as fatigue accumulates, potentially resulting in the prescription of relative loads (%1RM) that are higher than intended. The 1RM has been shown to be highly sensitive to RT-induced fatigue. For example, Häkkinen and Pakarinen [17] reported a 10.3% reduction in squat 1RM during a single session consisting of 20 sets of one repetition at 100% 1RM. Similarly, Hugues et al. [18] observed a 10.1% decrease in back squat 1RM 24 h after a RT session comprising five sets of squats to muscular failure at 70% 1RM. In line with these findings, Senturk et al. [19] demonstrated an 8.7% decrease in deadlift 1RM immediately after performing five sets of the exercise to failure at 70% 1RM. Collectively, these results indicate that relying on a pre-fatigue 1RM would have substantially overestimated the athletes’ actual performance capacity, potentially leading to the prescription of relative loads (%1RM) higher than intended. In this context, lifting velocity—which has also proven to be sensitive to fatigue [20, 21]—may offer a more practical alternative for estimating 1RM under fatigued conditions. Therefore, the key question arises as to which provides a more valid reference for individualizing loads when athletes experience RT-induced fatigue: direct 1RM testing performed in a non-fatigued state or velocity-based methods applied under fatigue. While direct 1RM testing benefits from greater test–retest stability, velocity-based approaches uniquely account for real-time fluctuations in neuromuscular readiness.

To our knowledge, no previous study has quantified how prediction errors of pre-session 1RM testing and velocity-based methods evolve across different levels of resistance training-induced fatigue. To address these gaps, the primary objective of this study was to determine whether pre-training direct 1RM testing or post-training load–velocity (L–v) profiling provides a more accurate estimate of the actual 1RM following RT-induced fatigue. Specifically, we compared both systematic biases and absolute errors between predicted and measured post-session 1RM under three conditions: control (rest), moderate fatigue (five sets at 70% 1RM with half of the maximum possible repetitions), and high fatigue (five sets at 70% 1RM to failure). We hypothesized that direct 1RM testing would provide greater predictive accuracy under control conditions, whereas velocity-based methods would yield more precise estimates following the moderate- and high-fatigue protocols.

## Materials and Methods

### Participants

An a priori power analysis (G*Power 3.1) was performed for the primary repeated-measures ANOVA comparing the post-session 1RM across the three experimental protocols. Assuming a medium effect size (*f* = 0.30), an alpha level of 0.05, a statistical power of 90%, three repeated measurements, a correlation of 0.60 among repeated measures, and a nonsphericity correction (ε) of 1, the minimum required sample size was estimated at 21 participants. To account for potential participant dropout, 27 young males were recruited for this investigation (mean ± standard deviation: age = 22.3 ± 2.1 years [range 19–28 years]; body mass = 78.8 ± 9.8 kg; stature = 178.6 ± 5.1 cm; HBD 1RM = 176.8 ± 20.9 kg; RT experience = 4.6 ± 2.2 years). Participants were recruited on a voluntary basis from the Faculty of Sports Sciences of Istanbul Gelişim University (Türkiye).

Inclusion criteria were being at least 18 years of age, having regularly incorporated the HBD exercise into their training routines, and demonstrating correct lifting technique during the familiarization session. Exclusion criteria included any musculoskeletal injury or physical conditions that could impair performance during the HBD exercise. Throughout the study period, participants were asked to refrain from engaging in any fatiguing lower-body workouts and to arrive well-rested for each testing session. Prior to data collection, detailed verbal information regarding the procedures was provided, and all subjects gave their written informed consent. The study was conducted at the Sport Performance Measurement and Evaluation Laboratory, Faculty of Sports Sciences, Istanbul Gelişim University, Istanbul, Türkiye. Participant recruitment and data collection were carried out between 29 March and 26 April 2024. The experimental protocol was reviewed and approved by the Ethics Committee of Istanbul Gelişim University (approval date: 15 March 2024; decision/approval no: 2024-04-68) and complied with the principles outlined in the Declaration of Helsinki.

### Study Design

A crossover study design was implemented to compare, under varying fatigue levels, the accuracy of %1RM-based load prescription between two strategies: pre-training direct 1RM testing and post-training L–v profiling. Each subject attended the laboratory on five occasions, with at least 48 h of rest between sessions. The first visit was used for familiarization, during which subjects performed the hexagonal barbell deadlift (HBD) exercise at maximal intended velocity with several submaximal loads. The second session involved an incremental loading protocol to establish each subject’s 1RM in the HBD. The order of the three remaining experimental sessions was counterbalanced across participants to ensure that each experimental condition was performed equally often in each ordinal position (first, second, and third), thereby minimizing potential order effects. In each experimental session, subjects completed a full incremental loading test at both the beginning and end of the session. The difference among the experimental sessions was the 30-min activity performed between the two loading tests: no training (control), moderate-fatigue RT, or high-fatigue RT. All testing took place in the same research facility under stable environmental conditions (~ 22–24 °C and ~ 60% humidity), supervised by the same investigator (DS), and scheduled at approximately the same time of day for each subject (± 1 h). This design allowed us to directly compare the accuracy of pre-session 1RM testing and post-session velocity-based methods in estimating the actual post-session 1RM under different levels of RT-induced fatigue.

### Familiarization Session (Session 1)

Body height and body mass were recorded using a stadiometer (Seca 202, Seca Ltd., Hamburg, Germany) and a segmental body composition analyzer (Tanita BC 418, Tokyo, Japan), respectively. Following a general warm-up consisting of light jogging and joint mobility exercises, subjects completed three repetitions of the HBD at four progressively heavier loads (30%, 50%, 70%, and 80% of their self-estimated 1RM), with 2 min of rest between sets. A 1-s pause with the bar resting on the floor was required between repetitions to avoid bouncing. Subjects were always instructed to perform the lifting phase as fast as possible while maintaining foot contact with the floor. All incremental loading tests in the present study were performed using this concentric-only execution technique.

In addition, subjects performed six continuous repetitions at 70% of their estimated 1RM using a touch-and-go execution technique, in which the barbell briefly contacted the floor between repetitions without a pause. Throughout the sets, subjects avoided scapular elevation, elbow or ankle flexion, and were consistently encouraged to lift the load with maximal intent. This touch-and-go execution, commonly used by the tested athletes in their training, was applied during the moderate- and high-fatigue protocols.

### Preliminary Testing Session (Session 2)

After performing the same general warm-up as during the familiarization session, subjects completed a specific warm-up consisting of one set of 8, 5, and 2 repetitions at 40%, 60%, and 80% of their self-estimated 1RM, respectively. Following this, the load was gradually increased until the 1RM in the HBD was determined. Load increments were adjusted based on mean velocity (MV): increases of 20–40 kg were applied when MV exceeded 0.80 m·s^−1^, 10–20 kg when MV was between 0.40 and 0.80 m·s^−1^, and 1–10 kg when MV fell below 0.40 m·s^−1^. The number of repetitions per load was also velocity-dependent: three for light loads (MV > 0.80 m·s^−1^), two for medium loads (MV = 0.50–0.80 m·s^−1^), and one for heavy loads (MV < 0.50 m·s^−1^). Rest intervals were standardized to 2 min for light, 3 min for medium, and 5 min for heavy loads. After reaching the 1RM, subjects rested for 5 min before performing a single set to failure at 70% of their newly established 1RM. The total number of repetitions completed in this set was later used to define the moderate-fatigue condition, in which subjects were instructed to perform half of that number.

### Experimental Testing Sessions (Sessions 3–5)

Each of the three experimental sessions began with the same general and specific warm-up protocol used in the preliminary testing. Subjects then completed the first incremental loading test of the session, which involved 3 repetitions at 30% of 1RM, 2 repetitions at 50% and 70% of 1RM, and 1 repetition at 80% and 90% of 1RM. After completing the 90% trial, subjects proceeded with single-repetition attempts, increasing the load until failure. The heaviest load lifted with correct technique was recorded as the 1RM. Rest intervals were standardized to 3 min for submaximal loads and 5 min between maximal attempts. The same incremental loading protocol was repeated at the end of the session.

The distinguishing feature across the three experimental conditions was the activity performed during the 30-min period between the pre- and post-session assessments: (i) control condition—passive rest for 30 min; (ii) moderate-fatigue condition—5 min of rest, followed by 5 sets of the HBD at 70% of 1RM with moderate effort (i.e., half the maximum number of repetitions established in the preliminary failure test), separated by 2-min rest intervals, and then 15 min of rest; and (iii) high-fatigue condition—5 min of rest, 5 sets of HBD to failure at 70% of 1RM with 2-min rest between sets, followed by 15 min of rest. These protocols were designed to create progressively greater levels of neuromuscular fatigue prior to the post-session assessment.

### Measurement Equipment and Data Analysis

The HBD exercise was executed using a standard 20-kg hexagonal bar loaded with calibrated weight plates ranging from 0.5 to 20 kg (Technogym, Italy). A validated linear position transducer (GymAware RS PowerTool; Kinetic Performance Technologies, Canberra, Australia) was secured to the right end of the bar using a Velcro strap to measure mean velocity (MV) during each repetition [22]. The velocity data were transmitted wirelessly via Bluetooth™ to a tablet device (iPad, Apple Inc., Cupertino, CA) running the GymAware application (version 2.8). The recorded data were automatically synced to the cloud and subsequently exported to Microsoft Excel (Microsoft Corp., Redmond, WA) for further processing and analysis.

Two velocity-based methods were employed to examine whether they could estimate the post-session 1RM more accurately than relying solely on the pre-session 1RM assessment. The first method involved modeling the post-session L–v relationship using four submaximal loads (50%, 70%, 80%, and 90% of 1RM), with the estimated 1RM derived from this relationship as the load corresponding to the MV recorded during the actual pre-session 1RM attempt. The second method first used the post-session MV recorded at each of the four submaximal loads (50%, 70%, 80%, and 90% of 1RM) together with the individual pre-session MV–%1RM relationship to estimate the corresponding relative load (%1RM). The post-session 1RM was then calculated for each load using the formula: load lifted (kg) × 100/estimated %1RM. In both methods, linear regression was used to model the relationships, as previous studies have reported that the linearity of the L–v relationship is preserved even under fatigue [23]. Only the repetition with the highest MV at each load was considered for analysis.

### Statistical Analyses

Descriptive statistics are presented as mean and standard deviation. The assumption of normality was assessed through visual inspection of Q–Q plots. Paired samples *t* test, Cohen's d effect size (ES), and Bland–Altman plots were used to assess the agreement between the directly measured post-session 1RM and each of the six predicted 1RM values. A two-way repeated-measures ANOVA was conducted on the absolute errors between the predicted and measured post-session 1RM, with “prediction method” (pre-session 1RM, post-session L–v profile, MV50%, MV70%, MV80%, and MV90%) and “training protocol” (control, moderate fatigue, and high fatigue) as within-subject factors. When significant main effects or interactions were observed, pairwise comparisons were performed using Bonferroni post hoc corrections. Sphericity was assessed using Mauchly’s test, and when the assumption was violated (*p* < 0.05), the Greenhouse–Geisser correction was applied. All statistical analyses were performed using SPSS software (version 25.0; IBM Corp., Armonk, NY, USA). The alpha level was set at 0.05.

## Results

**Fig. 1:**
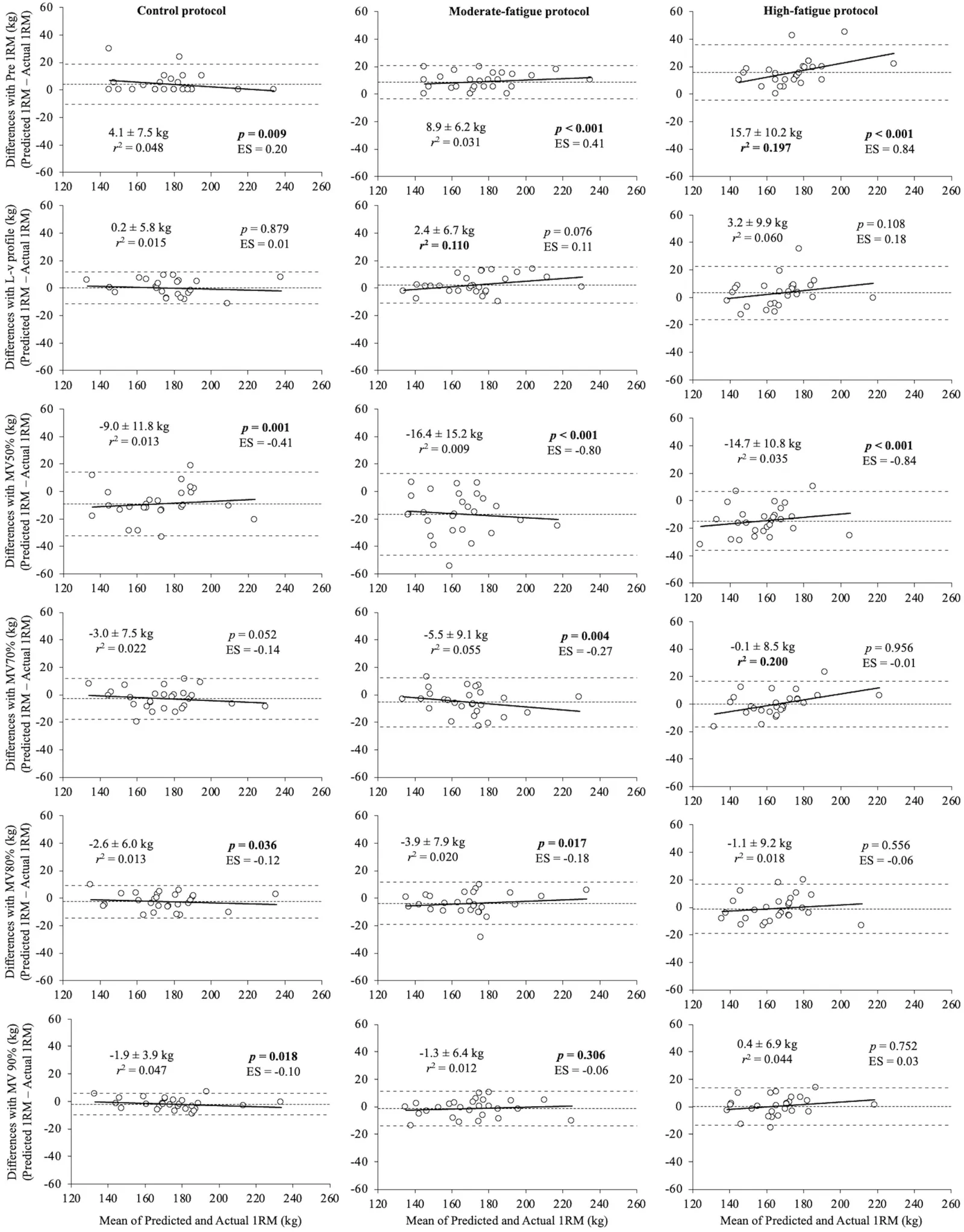
Agreement between the directly measured post-session 1RM and each of the six predicted 1RM values under the control (left column), moderate-fatigue (middle column), and high-fatigue (right column) protocols. Each panel shows a Bland–Altman plot with the mean bias (short dashed line), limits of agreement (± 1.96 SD, long dashed lines), and a continuous regression line to assess the presence of heteroscedasticity (*r*^2^). Paired samples *t* test results and corresponding ES values (Cohen’s d) are also reported. Bolded statistics indicate significant differences between the predicted and actual post-session 1RM (*p* < 0.05) and the presence of heteroscedasticity (*r*^2^ > 0.10). ES: effect size; MV: mean velocity; SD: standard deviation; 1RM: one-repetition maximum

All 27 enrolled participants completed all study procedures, and no participants or observations were excluded from the statistical analyses. Figure 1 displays the Bland–Altman plots assessing the agreement between the directly measured post-session 1RM and the six predicted values. The pre-session 1RM (9.5 ± 9.4 kg) and MV50% (− 13.4 ± 13.0 kg) substantially over- and underestimated the actual 1RM, respectively, whereas smaller errors were observed for the post-session L–v profile (1.9 ± 7.7 kg), MV70% (− 2.8 ± 8.6 kg), MV80% (− 2.5 ± 7.8 kg), and MV90% (− 0.9 ± 5.9 kg). For the pre-session 1RM, the degree of overestimation increased progressively with greater fatigue (control = 4.1 ± 7.5 kg; moderate fatigue = 8.9 ± 6.2 kg; high fatigue = 15.7 ± 10.2 kg). In contrast, no systematic trend in the magnitude of the bias was observed across protocols for any of the velocity-based methods.

**Fig. 2:**
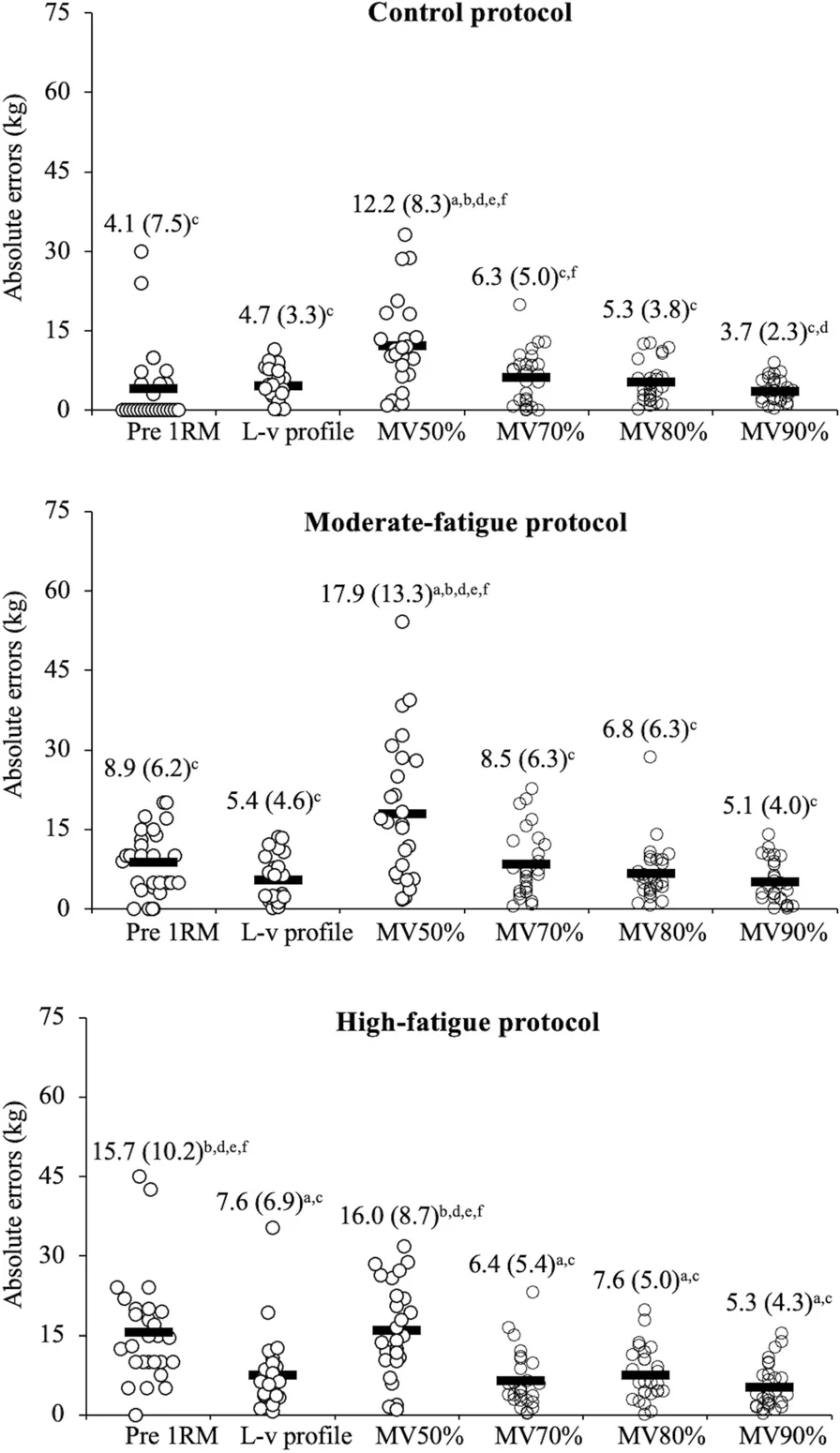
Absolute errors between the predicted and measured post-session 1RM for each prediction method under the control (upper panel), moderate-fatigue (middle panel), and high-fatigue (lower panel) protocols. Data are presented as mean ± SD with individual data points overlaid. **a** Significantly different from pre-session 1RM; **b** significantly different from post-session L–v profile; **c** significantly different from MV50%; **d** significantly different from MV70%; **e** significantly different from MV80%; **f** significantly different from MV90%. MV: mean velocity; SD: standard deviation; 1RM: one-repetition maximum

The ANOVA applied to the absolute errors revealed a significant main effect of prediction method (*F* = 26.3, *p* < 0.001). The methods were ranked from least to most accurate as follows: MV50% (15.4 ± 10.5 kg) < pre-session 1RM (9.5 ± 9.4 kg) < MV70% (7.1 ± 5.6 kg) = MV80% (6.5 ± 4.9 kg) = post-session L–v profile (5.9 ± 5.2 kg) < MV90% (4.7 ± 3.7 kg). The main effect of training protocol was also significant (*F* = 9.4, *p* < 0.001) due to the lower errors in the control protocol (6.0 ± 6.1 kg) compared to the moderate-fatigue protocol (8.7 ± 8.4 kg; *p* = 0.004) and high-fatigue protocol (9.8 ± 8.2 kg; *p* < 0.001). The prediction method × training protocol interaction also reached statistical significance (*F* = 3.9, *p* = 0.005), primarily driven by the pre-session 1RM method, whose prediction errors more than doubled under moderate fatigue (8.9 ± 6.2 kg) and more than tripled under high fatigue (15.7 ± 10.2 kg) compared to the control condition (4.1 ± 7.5 kg), while the errors for the other methods remained relatively stable across protocols (Fig. 2).

## Discussion

This study was designed to demonstrate that relying on a pre-session 1RM to prescribe training intensity (%1RM) may be less accurate than using real-time velocity-based measures under RT-induced fatigue. This expectation was based on the assumption that a pre-session 1RM does not account for the athlete’s neuromuscular state after accumulating fatigue during the session, whereas velocity-based measures are sensitive to fatigue and can offer a more responsive and individualized estimate of actual strength capacity. This hypothesis was confirmed by the systematic overestimation of post-session 1RM values when relying on the baseline pre-session 1RM, with the magnitude of error increasing in parallel with the level of RT-induced fatigue (high-fatigue protocol > moderate-fatigue protocol > control protocol). In contrast, smaller absolute errors were observed when using the post-session L–v profile and MV values obtained with heavier loads (70–90% 1RM). Among the velocity-based strategies tested, MV90% and the L–v profile emerged as the most effective and are, therefore, recommended for practitioners aiming to prescribe training loads more accurately under conditions of RT-induced fatigue.

Our first hypothesis was that direct 1RM testing would provide greater predictive accuracy than velocity-based measures under the control condition (two 1RM tests separated by 30 min). This expectation was grounded in the excellent test–retest reliability consistently reported for the actual 1RM [4] and in previous findings showing limited accuracy of velocity-based methods for predicting 1RM in free-weight lower-body exercises [24, 25]. However, this hypothesis was practically rejected, as the pre-session 1RM proved more accurate only when compared to MV50%, with no significant differences in prediction accuracy (absolute errors) relative to the L–v profile, MV70%, MV80%, or MV90%. A key finding was that, even in the control condition, the post-session 1RM was significantly lower than the pre-session value (176.0 vs. 180.1 kg), indicating that the initial 1RM assessment itself induced sufficient fatigue to impair maximal strength 30 min later. This observation has important practical implications, because it suggests that prescribing relative loads (%1RM) immediately after a direct 1RM assessment may inadvertently result in higher-than-intended training intensities, even in the absence of additional RT. Collectively, these results indicate that real-time velocity-based measures offer a similarly accurate but less time- and fatigue-demanding strategy for prescribing training loads during HBD sessions compared with relying on a pre-session direct 1RM. However, future studies should examine whether a direct 1RM determined in a separate session (without subsequent maximal testing on the same day) could serve as a more precise reference point for training load prescription.

Our second hypothesis was that velocity-based measures would outperform pre-session direct 1RM testing in predicting actual strength capacity under RT-induced fatigue conditions. This expectation was based on the unique ability of lifting velocity to reflect the athlete’s neuromuscular state in real time [26, 27]. The hypothesis was confirmed in both the moderate- and high-fatigue protocols, with the differences in predictive accuracy being particularly pronounced under the high-fatigue condition. Compared with the most accurate velocity-based method (MV90%), the absolute errors derived from the pre-session 1RM were nearly twice as large in the moderate-fatigue protocol and almost threefold greater in the high-fatigue protocol. From a practical perspective, these differences are not trivial. Under the high-fatigue protocol, relying on the pre-session 1RM resulted in a mean absolute error of approximately 16 kg, compared with only about 5 kg when using the MV90% method. Consequently, prescribing training loads based on a pre-session 1RM could result in athletes lifting substantially heavier relative loads than intended. Such discrepancies are likely to increase the physiological demands of the session, accelerate fatigue accumulation, reduce the number of repetitions that can be completed, and ultimately compromise the intended training stimulus. Therefore, when athletes are exposed to RT-induced fatigue, monitoring fluctuations in lifting velocity offers a more precise means of determining the actual %1RM being used than relying on a pre-fatigue 1RM. While it remains debatable whether training loads should be systematically adjusted downward as fatigue accumulates and lifting velocity declines, velocity-based monitoring nonetheless provides coaches with greater control over the actual stimulus imposed on the athlete—an essential aspect for establishing a clear dose–response relationship in RT [28].

This study presents several limitations that should be acknowledged. First, the sample consisted exclusively of young, resistance-trained men, which restricts the generalizability of the findings to women, untrained individuals, or elite athletes. While the direct assessment of 1RM has been shown to be reliable in both novice and experienced lifters [4], it is plausible that the L–v profile may be less reliable in novices, which could make one approach more advantageous than the other depending on the athlete’s profile. Second, only the HBD was investigated. While this exercise offers advantages in terms of safety and reduced technical complexity compared with other multi-joint free-weight movements, the results may not fully translate to exercises with different biomechanical demands or greater bar-path variability, such as the conventional deadlift or back squat. Third, the fatigue protocols were restricted to sets at 70% 1RM performed either to failure or to half of the maximal possible repetitions. Future studies should also investigate other common forms of fatigue frequently experienced by athletes—such as those resulting from cumulative training load, congested competition schedules, sleep restriction, travel, or psychological stress—to determine whether velocity-based strategies continue to outperform non-fatigued 1RM testing under these conditions. Finally, because all velocity measurements were obtained using a linear position transducer (GymAware RS PowerTool), commonly considered the gold standard for velocity-based training [22], the accuracy of the velocity-based methods may not be directly transferable to devices with lower measurement precision. Addressing these limitations will help clarify the external validity of our findings and refine practical recommendations for load prescription across a wider range of athletes and training contexts.

## Conclusions

When athletes train under fatigue, prescribing loads based on a pre-session 1RM may lead to systematic overestimations of intensity (%1RM), as this approach does not account for the athlete’s actual neuromuscular state during or after the session. In contrast, velocity-based methods appear to provide more fatigue-resilient estimates of 1RM, as lifting velocity is sensitive to RT-induced fatigue and reflects real-time changes in neuromuscular performance. However, not all velocity-based methods perform equally well. Predictions based on light loads (e.g., MV50%) tend to underestimate actual 1RM, likely because fatigue impacts movement velocity at low loads more than it affects maximal strength—an effect that diminishes as the load used to assess velocity increases.

Among the velocity-based strategies examined, the MV90% and the L–v profile appeared to provide the most accurate estimates of 1RM under fatigue. The MV90% method involves measuring MV with a heavy load (~ 90% 1RM) and applying it to the individual %1RM–MV relationship established in a prior test. While highly precise, it requires a previous 1RM assessment and assumes that this individual relationship remains stable over time. In contrast, the L–v profile does not require prior maximal testing if the minimal velocity threshold (MVT) is known, but it relies on recording velocity at a minimum of two submaximal loads within the same session to estimate 1RM. Despite their respective limitations, both methods may offer practical advantages over traditional load prescriptions when fatigue compromises the validity of pre-session 1RM values. These findings, however, should be interpreted within the context of resistance-trained young males performing the HBD, and further research is needed to determine whether they generalize to other populations and resistance exercises.

## Data Availability

The datasets generated and/or analyzed during the current study are available from the corresponding author upon reasonable request.
